# Caries and Socioeconomic Factors in Adults (19–60 Years Old): An Updated Systematic Review of Observational Studies

**DOI:** 10.3390/ijerph23010112

**Published:** 2026-01-16

**Authors:** Maria Aparecida Gonçalves de Melo Cunha, Alex Junio Silva da Cruz, Carolina Martins-Pfeifer, Simone de Melo Costa, Mauro Henrique Nogueira Guimarães de Abreu

**Affiliations:** 1 Graduate Program in Dentistry, School of Dentistry, Universidade Federal de Minas Gerais, Belo Horizonte 31270-901, MG, Brazil; magmc2010@ufmg.br (M.A.G.d.M.C.); aame0590@ufmg.br (A.J.S.d.C.); 2 Center for Integrative Global Oral Health, School of Dental Medicine, University of Pennsylvania, Philadelphia, PA 19104, USA; carolcm10@gmail.com; 3 Department of Dentistry, Universidade Estadual de Montes Claros, Montes Claros 39401-089, MG, Brazil; smelocosta@gmail.com; 4 Department of Community and Preventive Dentistry, School of Dentistry, Universidade Federal de Minas Gerais, Belo Horizonte 31270-901, MG, Brazil

**Keywords:** adults, caries, socioeconomic status, epidemiology

## Abstract

**Highlights:**

**Public health relevance—How does this work relate to a public health issue?**

- Dental caries in adults, 19–60 years old, is a major public health issue linked to social inequalities, disproportionately affecting vulnerable populations.
- This review synthesizes global evidence on how social determinants like income, education, and occupation contribute to these oral health disparities.

**Public health significance—Why is this work of significance to public health?**

- This work confirms that adults with lower socioeconomic status face higher disease risk and greater subsequent tooth loss from untreated caries.
- Oral health inequalities are not static but are shown to be the cumulative result of social and economic disadvantage experienced across the life course.

**Public health implications—What are the key implications or messages for practitioners, policy makers and/or researchers in public health?**

- Reducing oral health inequalities requires a policy shift from individual behavioral interventions to addressing upstream social and economic determinants.
- Effective strategies must integrate oral health into broader social policies and strengthen universal access to comprehensive preventive and restorative dental care.

**Abstract:**

Dental caries remains a major global public health problem characterized by pronounced social inequalities. This study aimed to identify, critically appraise, and synthesize the most recent evidence on the relationship between socioeconomic indicators and dental caries among adults aged 19–60 years, providing an updated systematic review that builds upon our previous reviews from 2012 and 2018. Reported following the PRISMA 2020 guidelines, we conducted a systematic search of eight electronic databases for observational studies published between March 2017 and April 2024 (PROSPERO: CRD42017074434). Two independent reviewers performed study selection, data extraction, and risk of bias assessment using the Newcastle–Ottawa Scale. Due to substantial methodological heterogeneity across the 22 included studies, a narrative synthesis was undertaken. The findings demonstrated a strong inverse association between socioeconomic position and caries experience. Lower income, lower educational attainment, and unemployment or employment in manual/unskilled occupations were associated with a higher overall caries experience. Advanced analytical approaches in recent studies, including life-course, reinforced that education and income are key contributors of these oral health inequalities, with persistent social disadvantage conferring the greatest risk. In conclusion, dental caries in adults aged 19–60 years is a social condition reflecting the cumulative effects of socioeconomic inequality across the life course. Addressing adult dental caries requires integrated approaches that combine clinical prevention with social and public policies aimed at reducing structural inequalities.

## 1. Introduction

Dental caries remains one of the most prevalent non-communicable diseases worldwide, affecting billions of individuals and imposing a substantial burden on people, health systems, and societies through pain, reduced quality of life, and considerable economic costs [1,2,3,4]. The distribution of this largely preventable condition is not random but follows a pronounced social gradient, disproportionately affecting vulnerable and disadvantaged populations [5,6]. Understanding the magnitude and mechanisms of these oral health inequalities continues to challenge public health research and policy.

Our previous systematic reviews, first published in 2012 [7] and subsequently updated with a meta-analysis in 2018 [8], established a solid evidence base in this field. Together, they synthesized data from numerous observational studies and consistently demonstrated an association between lower socioeconomic position—measured by education, income, and occupation—and greater severity of dental caries in adults [7,8]. The 2018 meta-regression further quantified this gradient, showing that populations with higher proportions of individuals in lower socioeconomic strata experience significantly greater burdens of decayed, missing, and filled teeth (DMFT) [8].

Since the 2018 update, the body of research has expanded. Despite broad recognition of the importance of addressing social determinants of health and shifting from disease prevention to health promotion, several studies have reported widening inequalities in the burden of oral diseases [9,10,11]. In Brazil, although overall levels of caries and edentulism have declined, improvements have been uneven, with more pronounced gains among White individuals and those with higher education, reflecting persistent inequities across subgroups [11]. In the United States, the burden of untreated caries in permanent teeth remains substantial, with widening cross-state disparities between 1990 and 2019 [10]. These findings highlight a more nuanced understanding of how social inequalities shape the distribution of dental caries, strengthening the rationale for an updated synthesis of the evidence.

This systematic review aims to identify, critically appraise, and synthesize the most recent literature on the relationship between socioeconomic indicators and dental caries in adults aged 19–60 years, thereby providing an updated assessment of the strength, consistency, and scope of this association.

## 2. Materials and Methods

This review followed the Preferred Reporting Items for Systematic Reviews and Meta-Analyses (PRISMA) 2020 guidelines [12] (Supplementary File S1). The protocol was registered with the International Prospective Register of Systematic Reviews (PROSPERO; CRD42017074434). This work updates two systematic reviews published in 2012 [7] and 2018 [8].

### 2.1. Eligibility Criteria

Inclusion and exclusion criteria were established using the Population, Exposure, Comparison, and Outcome (PECO) framework:

Population (P): Adults aged 19–60 years.

Exposure (E): Any validated measure of socioeconomic status (SES), including individual-level indicators (e.g., educational attainment, income, occupational status) or community-level indices (e.g., Gini coefficient).

Comparison (C): Groups with different levels of SES.

Outcome (O): Dental caries experience, measured by validated clinical metrics such as the Decayed, Missing, and Filled Teeth/Surfaces (DMFT/S) index, prevalence of untreated decay, or the presence of root caries.

### 2.2. Search Strategy

A comprehensive search was conducted in the following electronic databases: MEDLINE (via PubMed), Cochrane Library (including the Cochrane Database of Systematic Reviews, Database of Abstracts of Reviews of Effects, Cochrane Controlled Trials Register, and Cochrane Review Methodology Database), Web of Science, Controlled-Trials Database, ClinicalTrials.gov (U.S. National Institutes of Health), the National Institute for Health and Care Excellence, and the Virtual Health Library.

The search strategy combined subject headings and free-text terms adapted from the 2018 review (Supplementary File S2). Eligible study designs included observational studies (cross-sectional, cohort, case–control, and ecological). Interventional designs (e.g., clinical trials) were also considered during screening. To ensure completeness, reference lists of all retrieved articles were manually screened. No restrictions were applied regarding language. The electronic search was performed independently by two reviewers (CCMP and MAGMC) and included studies published from March 2017 up to April 2024.

### 2.3. Study Selection and Data Extraction

Search results were imported into EndNote 20 (Clarivate, 2013) for management. Following a calibration exercise on 10% of the records (inter-rater reliability, Kappa = 0.778), two reviewers (MAGMC and SMC) independently screened all titles and abstracts. We included studies in the full-text review if they enrolled participants aged 19–60 years and assessed the association between at least one socioeconomic indicator and dental caries, irrespective of whether socioeconomic status was the primary exposure. Studies with broader age ranges were included only if data for the 19–60-year age group were reported separately or could be clearly extracted. The full texts of 139 potentially eligible articles were subsequently retrieved and assessed for final inclusion. Any discrepancies were resolved through consensus or, if necessary, consultation with a third reviewer.

Data were extracted independently and in duplicate by two reviewers using a standardized, pre-piloted data extraction form. The following information was extracted from each study: (1) study characteristics (e.g., authors, year, country); (2) methodological details (e.g., study design, sample size); (3) participant demographics (e.g., age, sex); (4) definitions and categorization of SES indicators; (5) caries assessment methodology (e.g., diagnostic criteria, clinical index used); and (6) primary outcomes, including adjusted effect estimates with 95% confidence intervals where reported.

### 2.4. Risk of Bias Assessment

Two reviewers (MAGMC and SMC) independently appraised the methodological quality of included studies using the Newcastle-Ottawa Scale (NOS) [13]. This tool was selected to ensure methodological consistency with our previously published systematic reviews on this topic in 2012 and 2018. While acknowledging that domain-based tools are now preferred in current systematic review guidance, the NOS remains a widely used instrument that allows for a transparent and comparable appraisal across the body of evidence synthesized in this review series [14]. Observational studies were assessed with the NOS adapted for case–control designs. The NOS rates study quality from 1 (very poor) to 9 (high) across three domains: selection of study participants, comparability of groups, and ascertainment of exposure and outcomes. All studies meeting the inclusion criteria were retained, regardless of NOS score. Disagreements were resolved by discussion.

### 2.5. Data Synthesis and Analysis

A narrative synthesis of the evidence was performed to summarize and interpret the findings from the included studies. The synthesis was structured thematically, with studies grouped according to the primary socioeconomic determinant examined, such as educational attainment, income, occupational status, or community-level indices.

Although a quantitative meta-analysis was initially planned, it was considered inappropriate because of substantial clinical and methodological heterogeneity across the evidence base. This heterogeneity was evident in three key domains. First, the operationalization of socioeconomic indicators varied widely, ranging from categorical income and educational levels to composite indices and life-course social mobility trajectories. Second, the definition and measurement of dental caries differed considerably, including the use of different clinical indices (DMFT/S), individual components (e.g., untreated decay), and specific conditions such as root caries. Third, the diversity of reported effect measures—including odds ratios, relative risks, mean differences, and regression coefficients derived from different analytical models—precluded statistically valid pooling of results.

In this update, we focused on describing and synthesizing the new evidence retrieved in the current search strategy (publications from 2017 to 2024). This approach allows for a clear delineation of recent methodological advancements and emerging epidemiological patterns.

## 3. Results

**Figure 1:**
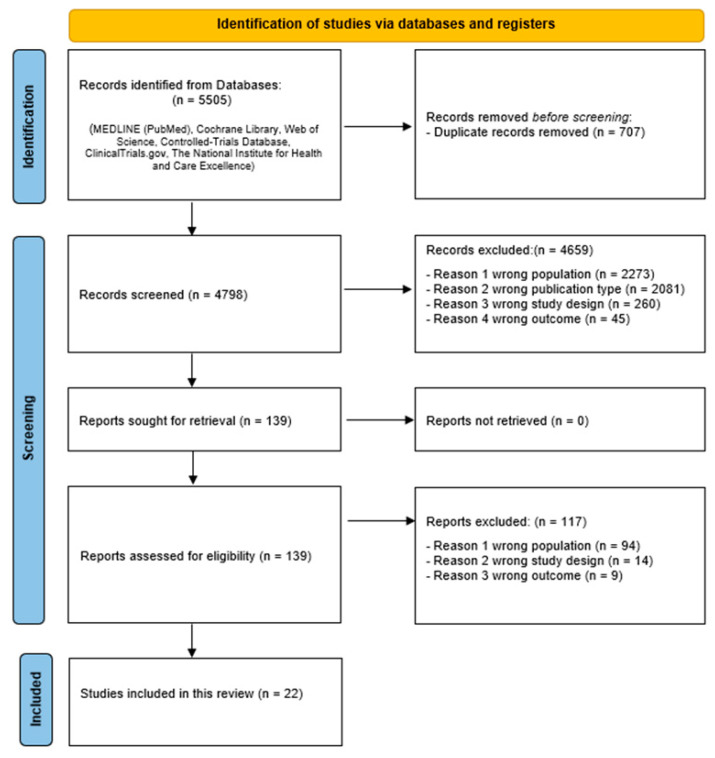
PRISMA flow diagram for systematic reviews.

In total, 5505 potentially relevant records were found. After removing duplicates, 4798 studies were read and 139 were selected for full-text analysis, 22 [15,16,17,18,19,20,21,22,23,24,25,26,27,28,29,30,31,32,33,34,35,36] of which were selected for inclusion in the qualitative synthesis (Figure 1).

### 3.1. Study Characteristics

The geographic scope of the evidence was extensive, reinforcing the global nature of this public health issue. Studies were conducted across Asia [16,19,21,25,26,27,32,36], Oceania [16], Europe [15,17,18,23,24,30,31], North America [20,29], and South America [22,28,33], in addition to one ecological study with a worldwide scope [34].

The included studies were predominantly of a cross-sectional design [15,17,18,19,21,22,25,27,28,30,33,36,37], with two ecological studies [32,34], one case–control study [20], and one analysis of repeated cross-sectional surveys [31]. The characteristics of the study populations were diverse, ranging from general national populations [19,20,29,31] to specific groups such as university students [36], formally employed workers [18,28], and dental patients [30]. Sample sizes varied substantially, from 84 individuals in a targeted study of working women [28] to 9812 participants in national health surveys [20], with two ecological studies analyzing data at the provincial or national level [32,34].

The primary outcome, dental caries, was measured using indices, most commonly the DMFT/S index and its individual components [15,16,17,18,19,22,23,24,25,26,30,31,32,33,34,36]. The outcome was also categorized as presence and absence [21,28]. Three studies assessed and reported data of root caries [18,19,27]. A feature across the included literature was the considerable heterogeneity in the definition and measurement of socioeconomic indicators, which encompassed income brackets, educational attainment levels, occupational hierarchies, and composite multi-item scales. The methodological quality of the 22 included studies was generally moderate to high, with scores on the NOS ranging from 5 to 8. One study was appraised at score of 8, nine studies scored 7, seven were rated as 6, and the remaining five studies scored 5. Supplementary Tables S1–S5 present the characteristics and extracted data from the primary included studies.

### 3.2. Data Synthesis

#### 3.2.1. Association Between Income and Dental Caries

A total of eleven studies investigated the association between income and dental caries experience. One study utilized a case–control design based on national survey data [20]. The measurement of income was highly heterogeneous across studies and included categories of household income, ratios relative to the federal poverty level, and subjective measures of financial strain [17,18,19,20,21,22,24,26,28,29]. Despite this methodological diversity, the direction of the association remained consistent—lower income was associated with a higher prevalence and severity of dental caries.

The association between lower income and untreated dental caries was strong. A large population-based study in Brazil found that individuals with a monthly income up to R$1500 (USD 187.02) were significantly more likely to have decayed teeth (OR 1.91; 95% CI 1.75–2.08) [22]. Similarly, a study of male dental students in Saudi Arabia reported that those with a family income below 10,000 SAR (USD 2664.60) had over three times the odds of having decayed teeth (OR 3.22; 95% CI 1.53–6.75) [36]. An analysis of US national survey data also revealed persistently high rates of decay among the lowest income groups over time [29].

In most multivariate analyses, low income remained a significant independent associated with higher caries experience after controlling for demographic and behavioral factors [19,20,21,22,26,36]. However, in two studies, the association was attenuated and no longer statistically significant in the final adjusted models [18,24]. One study did not perform a multivariate analysis to control for confounding [28].

Three studies specifically addressed root caries [18,19,27]. A study in Spain [18] found that workers with the lowest monthly income (≤€1200) had a significantly higher prevalence of root caries in bivariate analysis (*p* < 0.05); however, this association did not remain statistically significant in the final multivariate logistic regression model. Similarly, Gao et al. (2018) [19] in China reported a borderline significant association between lower annual household income and root caries in the 35–44-year-old age group (OR 0.96; 95% CI 0.93–1.00, *p* = 0.05). Supplementary Table S1 present the characteristics and extracted data from the primary included studies.

#### 3.2.2. Association Between Educational Attainment and Dental Caries

Sixteen studies provided data on the association between educational attainment and dental caries [15,16,17,18,19,20,21,22,24,26,27,28,31,32,35,36]. The measurement of education varied across studies, including years of schooling (e.g., ≤8 years vs. >8 years), highest level attained (e.g., primary, secondary, university), and composite low/medium/high categories.

The relationship between lower education and untreated dental decay was noted. In a large Brazilian study, adults with up to 8 years of schooling had significantly higher odds of having decayed teeth (OR 1.32; 95% CI 1.12–1.56) compared to their more educated counterparts [22]. This was strongly corroborated by a study in Thailand, where individuals with primary education or lower had almost twice the odds of having untreated caries (OR 1.97; 95% CI not reported, *p* = 0.007) in the fully adjusted model [21]. A 20-year trend analysis in Lithuania found, for adults 35–44 years old, that having more than secondary school was a significant protective determinant for dental caries, measured by decayed surfaces (IRR 0.50 (95% CI: 0.39–0.64) [31].

Lower educational attainment was also consistently linked to a worse general caries experience. Nogueira et al. (2019) reported that having 8 or fewer years of education was associated with a greater likelihood of having more than 16 DMFT (OR 1.51; 95% CI 1.35–1.69) and, most strikingly, more than double the odds of having more than four missing teeth (OR 2.13; 95% CI 1.90–2.38) [22]. In Norway, bivariate analysis showed that individuals with only secondary schooling had a significantly higher mean number of carious surfaces than those with a university education (*p* < 0.001) [17]. In contrast, one study in India reported a lower risk of caries for those with primary education in the multivariate model (RR 0.80 [95% CI: 0.70–0.91] *p* = 0.001) [26].

A national survey in China found that a high education level was a significant protective factor against root caries in middle-aged (35–44 years) adults (OR 0.63 [95% CI 0.56–0.71] *p* < 0.001) [19]. Similarly, a study among Spanish workers found a significant bivariate association, with the highest prevalence of root caries among those with only primary studies (*p* < 0.05). However, this association lost its statistical significance in the final multivariate logistic regression model, suggesting its effect may be mediated by other factors [18]. Supplementary Table S2 present the characteristics and extracted data from the included studies.

#### 3.2.3. Association Between Occupational Status and Dental Caries

Six studies examined the relationship between occupational status and dental caries [15,21,26,28,35,36] (Supplementary Table S3). Heterogeneity in how occupational status was defined and measured, ranging from hierarchical classifications (e.g., professional, skilled, unskilled) to categorical types (e.g., agriculture, business) and simple employment status (employed vs. unemployed) was observed.

In an Italian population, Arrica et al. (2017) found that unemployed individuals or housewives had a significantly higher DMFT compared to technicians, clerks, and professionals (*p* < 0.01) [15]. In the multivariate analysis, being unemployed/a housewife was associated with a progressively higher risk of being in a worse DMFT category, with the relative risk ratio (RRR) reaching 3.19 (95% CI: 2.10–4.84) for the highest caries category (15–28 DMFT) [15]. Similarly, in India it was reported that unskilled workers (RR 1.37 [95% CI: 1.04–1.82] *p* = 0.03) had a significantly higher risk of dental caries compared to semiprofessional/professionals [26]. In Thailand, while bivariate analyses showed significant associations for several occupational groups, these relationships were no longer statistically significant in the final multivariate model that adjusted for income, education, and other confounders [21]. Similarly, a study of working women in Colombia did not find a statistically significant bivariate association between the type of role at their university (e.g., professor, general services) and the presence of dental caries (*p* = 0.635) [28]. It is noteworthy that the studies reporting an attenuated or non-significant association for occupational status both received moderate quality scores (NOS = 6).

#### 3.2.4. Association Between Socioeconomic Status and Dental Caries

Five studies explored the relationship between a measure of SES and dental caries outcomes in adults [25,26,28,30,33] (Supplementary Table S4). These studies were conducted in India, Colombia, Croatia, and Brazil, all employing cross-sectional designs. The measurement of SES was a point of heterogeneity, ranging from standardized multi-component scales like the Kuppuswamy’s Scale in India, to self-assessed household economic status in Croatia, and a life-course social mobility approach in Brazil. Despite these varied methodologies, the majority of the evidence confirms that lower SES is associated with a greater burden of dental caries.

Three studies utilized composite SES scales. In India, Gijwani et al. (2020) used Kuppuswamy’s Scale and found a statistically significant bivariate association (*p* = 0.01), with the highest mean DMFT scores observed in the upper-lower group [25]. Another study in India by Singla et al. (2020), also using Kuppuswamy’s Scale, reported that compared to the highest SES group (Upper), the lowest SES groups appeared to have a reduced risk of caries, but the findings were no longer statistically significant in the final model [26].

The two studies employing alternative measures of SES provided strong evidence for a social gradient. In a Croatian population, Bukmir et al. (2022) [30] used a self-assessed measure of household economic status. Their multivariate regression model revealed a highly significant and strong association: as self-assessed economic status increased, the number of untreated decayed teeth significantly decreased (β = −1.296; *p* < 0.001) [30]. Celeste et al. (2024), who analyzed social mobility over the life course, provided a dynamic perspective [33]. Their findings demonstrated the cumulative impact of socioeconomic disadvantage. Individuals in the “persistently lower” socioeconomic group (low SEP in both childhood and adulthood) had the highest prevalence of both missing teeth (86.7%) and untreated decayed teeth. Conversely, those in the “persistently higher” group had the lowest prevalence, highlighting a gradient shaped by life-long socioeconomic trajectories (*p* < 0.01).

#### 3.2.5. Association Between Collective Indicators and Dental Caries

Nine studies provided data on the association between collective-level indicators and dental caries [17,21,23,26,28,30,31,32,34] (Supplementary Table S5). These indicators can be broadly categorized into two main types: (1) Geographic disparities within countries, such as urban-rural residence and regional differences, and (2) Macro-economic indicators in ecological studies, such as Gross National Income (GNI).

Eight studies examined disparities based on geography within a country. The most common comparison was between urban and rural populations, with a majority of studies reporting worse caries outcomes in rural areas. In Norway, individuals in rural municipalities had a significantly higher mean number of carious surfaces compared to their urban counterparts (*p* < 0.001) [17]. Similarly, a multivariate regression in Croatia revealed that living in a rural area was a associated factor for a higher number of decayed teeth (β = −2.008, indicating urban residence was protective (*p* < 0.001) [30]. In contrast, it was noted a higher, though not statistically significant, prevalence of caries among urban Colombian working women (*p* = 0.267) [28].

A worldwide study classified 170 countries by Gross National Income (GNI) and found a significant association between a country’s income level and the mean DMFT of its population (*p* = 0.004) [34]. This confirms that, on a global scale, national wealth is a powerful determinant of a population’s overall caries experience. However, an ecological study within Iran by Tahani et al. (2024) (32) analyzed data at the provincial level and found that GNI per capita was not significantly associated with mean DMFT or edentulousness in either simple or multiple linear regression models [32].

## 4. Discussion

This update was undertaken because, since the publication of our previous reviews, the evidence base has expanded not only quantitatively but also qualitatively, incorporating new populations, outcomes, and analytical approaches that cannot be adequately represented by referring solely to earlier syntheses. Our synthesis of 22 new studies, spanning multiple continents and varied healthcare systems, shows that lower SES is consistently associated with a greater burden of dental caries. A key insight from this updated evidence is the differential impact of SES on the components of the DMFT/S indices. The association is more prominent and most consistent for untreated decayed teeth, suggesting that social disadvantage is associated with both an elevated risk of disease onset and constrained access to timely, restorative dental care, thereby accelerating tooth loss [22,30,38]. This accumulating body of evidence also reflects a conceptual maturation of the field, as recent research employs more sophisticated theoretical and analytical frameworks that move beyond describing inequality to elucidating its complex and multifactorial origins [15,33].

These findings are largely consistent with the conclusions of our 2012 systematic review [7] and 2018 meta-analysis [8], but they add depth and nuance. The relative weight of individual socioeconomic indicators appears to be context-dependent. While many studies confirmed the independent effects of both income and education, research by Amornsuradech and Vejvithee (2019) [21] in Thailand found that education remained a strong predictor of untreated caries even after controlling for income, suggesting that mechanisms such as health literacy may be paramount. This social gradient was not confined to high-income nations [17]; consistent associations were also reported in middle-income settings across South America and Asia [21,22,25]. The focus on root caries in some studies [18,19,27] also contributes to a broader understanding, confirming that socioeconomic gradients are present in this often-overlooked oral condition—a finding of particular relevance given global population aging and increased tooth retention [11,39].

The analyzed data from included studies support for conceptual models that frame oral health within the broader social determinants of health framework [40,41]. Socioeconomic position acts as an “upstream” structural determinant, shaping an individual’s exposure to a cascade of more proximal, or “intermediary,” risk factors over their lifetime [42,43]. This review provides evidence for some mediating pathways. Social capital represents a critical pathway; an emerging area of research suggests that the chronic stress and lower social cohesion associated with socioeconomic disadvantage can directly impact health-promoting behaviors [22,44,45]. Furthermore, the findings related to missing teeth and untreated decay highlight the role of healthcare systems, illustrating the inverse care law whereby those with the greatest need have the least access to appropriate services [46]. The study by Celeste et al. (2024) [33], which analyzed social mobility, provides evidence that oral health in adulthood appears to reflect cumulative socioeconomic exposures across the life course, with ‘persistently lower’ socioeconomic trajectories conferring the greatest risk.

The primary strength of this review lies in its comprehensive synthesis of the available evidence published from 2017 to 2024. Methodological quality was assessed using the NOS, and to integrate risk of bias into the synthesis, we compared studies with higher methodological quality (NOS ≥ 7) [15,17,18,20,22,25,26,27,30,34], with those with moderate/low quality scores (NOS ≤ 6) [16,19,21,23,24,28,29,31,32,33,35,36], as detailed in Supplementary Tables S1–S5. Across studies, the direction of the association between lower socioeconomic status and a greater burden of dental caries was consistent, irrespective of NOS score. However, qualitative differences were observed in the strength and precision of effect measures. Studies with higher NOS scores more frequently reported robust and statistically significant associations, particularly in fully adjusted multivariable models, whereas several attenuated or non-significant findings were observed among studies with moderate methodological quality, often characterized by limited confounder adjustment or more restricted study populations. This pattern indicates that socioeconomic inequalities in dental caries are a consistent finding, and that variability in effect estimates is partly attributable to differences in methodological rigor rather than true inconsistency in the underlying association.

Several limitations inherent to the primary literature should be acknowledged. The most significant limitation is the heterogeneity in outcome reporting—using odds ratios, risk ratios, *p*-values, or correlation coefficients—which precluded quantitative synthesis through meta-analysis. Although alternative age classifications for adulthood exist in the literature [47], the consistent application of the 19–60-year age range in this updated review preserved methodological consistency and comparability over time. In addition, the evidence base is composed exclusively of observational studies, which limits causal inference. While the pathway from social disadvantage to poor health is well-established, the potential for confounding from unmeasured variables remains.

The implications of this body of evidence for public health policy and practice should be highlighted. Our findings demand a strategic pivot toward equity-oriented, intersectoral policies that address the upstream social and economic determinants of oral health [48]. This includes: (1) integrating oral health considerations into broader social policies aimed at reducing income inequality and improving educational attainment; (2) strengthening universal health coverage by critically examining not only eligibility but also effective access, given that structural barriers, including inadequate provider reimbursement and the geographic maldistribution of dental practices, may undermine the intended benefits of public programs; and (3) adopting a “common risk factor approach” that targets determinants, such as sugar consumption, that are shared with other major chronic diseases [48,49]. These recommendations align directly with the World Health Organization’s global agenda on addressing the social determinants of health and achieving universal health coverage [50].

To build upon this evidence and better inform effective interventions, several directions for future research emerge. More prospective cohort studies are needed to elucidate the dynamic effects of social mobility and cumulative disadvantage over life course. Multilevel modeling is also essential to disentangle the respective contributions of individual- and contextual-level factors [21]. Also, there is a pressing need for high-quality, equity-oriented intervention research that evaluates the effectiveness of specific upstream policies (e.g., fiscal, educational, or service-delivery interventions) in reducing oral health inequalities among adults. Finally, the development of consensus-based, standardized methods for measuring and reporting socioeconomic indicators would greatly enhance the comparability of future studies.

## 5. Conclusions

Among adults aged 19–60 years old, dental caries is a social condition reflecting the cumulative effects of socioeconomic inequality across the life course. This updated review confirms that adverse socioeconomic indicators are still associated with greater caries experience. Addressing adult dental caries requires integrated approaches that combine clinical prevention with social and public policies aimed at reducing structural inequalities.

## Data Availability

All data generated or analyzed during this study are included in this published article.

## Supplementary Materials

The following supporting information can be downloaded at: https://www.mdpi.com/article/10.3390/ijerph23010112/s1, Supplementary File S1: PRISMA checklist; Supplementary File S2: Search strategy for each electronic database; Supplementary Table S1: Study characteristics and results reported of the indicator income; Supplementary Table S2: Study characteristics and results reported of the indicator education; Supplementary Table S3: Study characteristics and results reported of the occupational status; Supplementary Table S4: Study characteristics and results reported of the socioeconomic status; Supplementary Table S5: Study characteristics and results reported of the collective indicators and other.

## Abbreviations

The following abbreviations are used in this manuscript:

DMFT	Decayed, Missing, And Filled Teeth
GNI	Gross National Income
NOS	Newcastle–Ottawa Scale
PECO	Population, Exposure, Comparison, and Outcome
PRISMA	Preferred Reporting Items for Systematic Reviews and Meta-Analyses
SES	Socioeconomic Status
