# Supplementary material for: Caries and Socioeconomic Factors in Adults (19–60 Years Old): An Updated Systematic Review of Observational Studies
Source: Int J Environ Res Public Health. 2026 Jan 16;23(1):112. doi: 10.3390/ijerph23010112 (PMC12840810; doi:10.3390/ijerph23010112)
Supplement: Supplementary file 1 [file ijerph-23-00112-s001.zip › ijerph-4055679-supplementary.pdf]

Supplementary Table S1. Study characteristics and results reported of the indicator income.

Authors, year, country	Study type	Sample in the analysis	Caries index	Effect measure	Income: categories of analysis Socioeconomic parameter (reference*)	Group with higher caries index	Bivariate Analysis p value	Multivariate analysis p value	NOS
Oscarson; Espelid; Jönsson (2017) Norway	Cross-sectional	932 adults (988 women) with at least one tooth, mean age 47.0 years (SD 15.3)	DMF-S, DF-S, DMF-T, DF-T, DT, Number-T Intact-T.<br>DS1-5 (Sum of enamel and dentine caries)<br>DS1-2 (Enamel caries)<br>DS3-5 (Dentine caries)	Mean, odds ratios (OR)	20% lowest<br>Medium low<br>Medium high<br>25% highest	20% lowest (Mean number of surfaces with primary caries: 4.6 [SD 6.0)	p<0.056	Household income was not included in the regression model	7
Saura-Moreno et al. (2017) Spain	Cross-sectional	458 workers aged 35-44 years	DF-S (Decayed, Filled Surfaces - Root Caries); Active Root Caries	Prevalence, means (SD); OR	≤ €1200/month; €1200-€3600/month; (> €3600/month*)	≤ €1200/month	p<0.05	Multivariate logistic regression model for DF-S (root) >0<br>≤ €1200/month (OR 4.0 [95% CI: 0.4-45.1] p=0.2645)<br>€1200-€3600/month (OR 3.1 [95%CI: 0.3-34.4] p=0.3668)	7
Gao et al. (2018) China	Cross-sectional, national survey	35-44 age group: 4410 participants (2197 males, 2213 females)<br>65-74 age group: 4431 participants (2222 males, 2209 females)	DR (Decayed Roots); FR (Filled Roots without primary or secondary caries); RCI (Root Caries Index - number of teeth with root caries lesions / number of teeth with gingival recession x 100%)	Prevalence, means (SD); OR	low, medium, high (reference not direct specified)	not direct specified	p values not reported	Annual household level<br>35-44-y-old (OR 0.96 [95%CI 0.93-1.00] p=0.05)<br>65-74-y-old (OR 1.00 [95%CI 1.00-1.00] p=0.60)	6
Zanella-Calzada et al. (2018) United States	Case-control	9812 participants from NHANES 2013-2014 (6122 cases with dental caries, 3690 controls without caries) split across three age groups, excluding 0-9 year olds due to lack of data:<br>Group 2 (10-19 years): 112 subjects (50 cases, 62 controls)<br>Group 3 (20-59 years): 7603 subjects (4551 cases,	Presence/absence of dental caries (binary: 1 = caries present, 0 = caries absent)	OR	Total household income; Total family income; Ratio of family income to poverty guidelines.		Not reported	**Age group 3**<br>Income was not included<br>**Age group 4**<br>Ratio of family income to poverty guidelines (OR 1.205 [CI 2.5%-97.5%: 1.112 – 1.308])	7

Authors, year, country	Study type	Sample in the analysis	Caries index	Effect measure	Income: categories of analysis Socioeconomic parameter (reference*)	Group with higher caries index	Bivariate Analysis p value	Multivariate analysis p value	NOS
		3052 controls)<br>Group 4 (60+ years): 2097 subjects (1521 cases, 576 controls)							
Amornsuradecs; Vejvithee (2019) Thailand	Cross-sectional	1518 adults aged 35-44 years from the 7th Thailand National Oral Health Survey (2012)	Number of teeth with untreated dental caries (0 or ≥1).	OR	Average income per month, categorized as 0-15,000 baht or (>15,000 baht*)	0-15,000 bah	p=0.028	(OR 1.345 [95%CI: not reported]; p= 0.315)<br>Model: SES variables adjusted for income, education, occupation, personal background, behavioral and access	6
Nogueira et al. (2019), Brazil	Cross-sectional	6051 adults aged 35-44 years	DMFT index (dichotomized by the median into <16 and >16),<br>number of decayed teeth (dichotomized by the<br>median into 0 and >0), and number of missing teeth (dichotomized by the median into <4 and >4).	OR	up to R$1,500 and (>R$1,500*)	up to R$1,500	Not reported	**Decayed>0**<br>(OR 1.91 [95%CI 1.75-2.08])<br><br>**Missing>4**<br>(OR 1.29 [95%CI 1.15–1.44])<br><br>**DMFT>16**<br>(income did not enter in the adjusted model)	7
Singla et al. (2020) India	Cross-sectional	780 adults aged 20-50 years-old	DMF-T; D-T; M-T; F-T	Mean (SD) Relative Risk (RR)	Monthly income (in Rupees): <10356; 10,357–20,714; 20,715–41,429; (>41,430*)	>41,430	Not reported	<10356 (RR 1.15 [95%CI: 0.91–1.44] p=0.05)<br>10,357–20,714 (RR 1.01 [95%CI: 0.81-1.24] p=0.96)<br>20,715–41,429 (RR 0.94 [95%CI: 0.82-1.08] p=0.38)	7
Beenackers et al. (2021), Netherlands	Secondary data analysis of a cross-sectional survey conducted in 2013	452 dentate adults aged 25-44 years (combined sample of 217 aged 25-34 and 235 aged 35-44)	DMF-S. Caries were assessed at the dentine threshold (D3).	OR; RR	Less than standard; (Standard*); 1-2 times standard; More than 2 times standard; Don't want to say	Not reported	Not reported	Negative binomial hurdle models for caries experience (having a DMFS >0 versus DMFS = 0)<br>**MODEL 1** (The first model explored the association between financial strain and	6

Authors, year, country	Study type	Sample in the analysis	Caries index	Effect measure	Income: categories of analysis Socioeconomic parameter (reference*)	Group with higher caries index	Bivariate Analysis p value	Multivariate analysis p value	NOS
								DMFS - adjusted for age, sex, educational level, and income)<br><br>Less than standard (OR 1.26 [95%CI: 0.35-4.55])<br>1–2 times standard (OR 1.08 [95%CI: 0.39-2.99])<br>>2 times standard (OR 1.29 [95%CI: 0.40-4.12])<br>Don't want to say (OR 1.73 [95%CI: 0.31-9.63])<br><br>Negative binomial hurdle models for caries experience (amount of DMFS in those having a DMFS >0)<br>**MODEL 1** (The first model explored the association between financial strain and DMFS- adjusted for age, sex, educational level, and income)<br><br>Less than standard (RR 1.24 [95%CI:0.88-1.73])<br>1–2 times standard (RR 1.17 [95%CI: 0.87-1.55])<br>>2 times standard (RR 1.08 [95%CI: 0.77-1.53])<br>Don't want to say (RR 1.27 [95%CI: 0.88-1.85])	
Jouhar et al. (2021) Saudi Arabia	Cross-sectional	185 male dental students and interns	DMF-T; D-T; M-T; F-T	OR	<10,000; 10,001–20,000; 20,000–30,000; >30,000. For the regression models: <10,000; (>10,000*)	Not reported	Not reported	Decayed (Yes/No)<br>(OR: 3.22 [95%CI 1.53–6.75])<br><br>Missing (Yes/No)<br>(OR: 0.80 [95%CI 0.40–	5

Authors, year, country	Study type	Sample in the analysis	Caries index	Effect measure	Income: categories of analysis Socioeconomic parameter (reference*)	Group with higher caries index	Bivariate Analysis p value	Multivariate analysis p value	NOS
								1.62])<br><br>Filled (Yes/No)<br>(OR: 2.08 [95%CI 0.93–4.68])	
Almario-Barrera; Concha-Sánchez (2022) Colombia	Cross-sectional	84 working women at a private university in Santander, Colombia	Presence/Absence of dental caries	Prevalence	1 salary; 2 salaries; 3-4 salaries; 5-6 salaries (salary= 877.803 SM-MLV)	Within those with dental caries: 1 salary (n=35; 48.6%)	p=0.165	Not performed	6
Borges; Krishnamurthy (2022) USA	Cross-sectional	Data from the National Health and Nutrition Examination Survey (NHANES). The exact sample sizes varied slightly for each of the five oral health indicators examined, based on data availability and survey design, but the total sample represents the US population.	Presence of caries in permanent or deciduous teeth	Prevalence and percentage change from baseline to follow-up period	Federal Poverty Level: <100%; 100-199%; 200-399%; 400-499%; 500%+	<100%	Distribution of OH 3.1: decay 35–44 years<br>Income<br><100 (1999-2004 [estimate: 49.4; Std er.: 3.196; 95% CI: 42.9–55.8) 2013–2016 (1999-2004 [estimate: 50.0; Std er.: 2.990; 95% CI: 43.9–56.1). Change: 1.21. p= 0.1789<br><br>100–199 (1999-2004 [estimate: 44.9; Std er.: 2.948; 95% CI: 39.0–50.9) 2013–2016 (1999-2004 [estimate: 38.6; Std er.: 3.255; 95% CI: 32.2–45.5). Change: -14.03. p=0.2024<br><br>200–399 (1999-2004 [estimate: 27.1; Std er.: 2.354; 95% CI: 22.4–31.9) 2013–2016 (1999-2004 [estimate: 28.2; Std er.: 2.648; 95% CI:	Not performed	5

Authors, year, country	Study type	Sample in the analysis	Caries index	Effect measure	Income: categories of analysis Socioeconomic parameter (reference*)	Group with higher caries index	Bivariate Analysis p value	Multivariate analysis p value	NOS
							23.1–33.9). Change: 4.06. p= 0.2197<br><br>400–499 (1999-2004 [estimate: 21.0; Std er.: 3.106; 95% CI: 14.7–27.4) 2013–2016 (1999-2004 [estimate: 14.2; Std er.: 3.144; 95% CI: 8.9–21.9). Change: -32.38. p= 0.3412<br><br>500 (1999-2004 [estimate: 8.1; Std er.: 1.864; 95% CI: 4.3–11.8) 2013–2016 (1999-2004 [estimate: 11.4; Std er.: 2.557; 95% CI: 7.1–17.7). Change: 40.74. p= 0.0258<br><br>Distribution of OH 4.1 permanent tooth extraction 45–64 years<br>Income<br><100 (1999-2004 [estimate: 89.9; Std er.: 2.083; 95% CI: 85.8–94.0) 2013–2016 (1999-2004 [estimate: 91.0; Std er.: 1691; 95% CI: 86.9–93.9) Change: 1.22. p= 0.4248<br><br>100–199 (1999-2004 [estimate: 92.9; Std er.: 1.309; 95% CI:		

Authors, year, country	Study type	Sample in the analysis	Caries index	Effect measure	Income: categories of analysis Socioeconomic parameter (reference*)	Group with higher caries index	Bivariate Analysis p value	Multivariate analysis p value	NOS
							90.4–95.5) 2013–2016 (1999-2004 [estimate: 83.4; Std er.: 1794; 95% CI: 79.4–86.7) Change: -10.23. p=0.0249<br><br>200–399 (1999-2004 [estimate: 81.1; Std er.: 2.179; 95% CI: 76.8–85.4) 2013–2016 (1999-2004 [estimate: 80.2; Std er.: 1859; 95% CI: 76.2–83.8) Change: -1.11. p= 0.4557<br><br>400–499 (1999-2004 [estimate: 75.8; Std er.: 2.588; 95% CI: 70.7–80.9) 2013–2016 (1999-2004 [estimate: 63.7; Std er.: 4639; 95% CI: 53.8–72.5) Change: -15.96. p= 0.0143.<br><br>500 (1999-2004 [estimate: 62.2; Std er.: 1.903; 95% CI: 58.5–66.0) 2013–2016 (1999-2004 [estimate: 53.9; Std er.: 2752; 95% CI: 48.2–59.4) Change: -13.34. p= 0.1870		

Risk of bias was assessed using the Newcastle-Ottawa (NOS) for observational studies (Wells et al. 2012) with scores for summarizing the multitude data. DMFT decayed missing filled teeth; DT decayed teeth; DS/DFS decayed (filled) surfaces; DMFS decayed missing filled surfaces; RDFS, RDS decayed (filled) surfaces root; DF decayed root; Mean (SD) standard deviation; (SE) standard error; RR risk ratio; 95%CI confidence interval; OR odds ratio; PR prevalence rate; R correlation coefficient.

Supplementary Table S2. Study characteristics and results reported of the indicator education.

Authors, year, country	Study type	Sample in the analysis	Caries index	Effect measure	Income: categories of analysis Socioeconomic parameter (reference*)	Group with higher caries index	Bivariate Analysis p value	Multivariate analysis p value	NOS
Arrica et al. (2017) Italy	Cross-sectional	480 adults (52.9% men, 47.1% women), aged 30-45 years	DMF-T and FS-T (Filled and Sound Teeth)	Chi-Square (χ2) Relative Risk Ratio (RRR), SE, (95% CI)	dichotomized as: (primary/secondary school*) vs. university degree	primary/secondary school	X2=25.70 (p<0.01)	3-23 FS-T (RRR 0.51 [95% CI: 0.33-0.79] p<0.001); 24-25 FS-T (RRR 0.48 [95% CI: 0.32-0.73] p<0.001); 26-27 FS-T (RRR 0.69 [95% CI: 0.50-0.95] p<0.001); 28 FS-T (base outcome)	8
He; Thomson (2018) China (Sichuan Province) and New Zealand	Secondary analysis of representative data from oral health surveys (2005 for China and 2009 for New Zealand)	Overall, 2,699 China (35-44 age group): 788 participants New Zealand (35-44 age group): 773 participants China (65-74 age group): 771 participants New Zealand (65-74 age group): 367 participants	DMF-T	Mean (SD)	primary, middle, tertiary	primary (statistically Signiant only for 35-44/64-75-y-old Chinese)	p<0.05	Not performed	5
Oscarson; Espelid; Jönsson (2017) Norway	Cross-sectional	932 adults (988 women) with at least one tooth, mean age 47.0 years (SD 15.3)	DMF-S, DF-S, DMF-T, DF-T, DT, Number-T Intact-T. DS1-5 (Sum of enamel and dentine caries) DS1-2 (Enamel caries) DS3-5 (Dentine caries)	Mean caries scores, odds ratios (OR)	Secondary school; High School; (University*)	High school (Mean number of surfaces with primary caries: 4.4 [SD 6.3)	p<0.001	Univariate analysis Sec. school (OR 1.2 [95%CI: 0.9-1.6] p=0.184) High school (OR 0.8 [95% CI 0.7-1.1] p= 0.168) Not included in the adjusted model	7
Saura-Moreno et al. (2017) Spain	Cross-sectional	458 workers aged 35-44 years	DF-S (Decayed, Filled Surfaces - Root Caries); Active Root Caries	Prevalence, means (SD); OR	Primary, secondary, (university*)	primary	p<0.05	Multivariate regression model for DF-S (root) >0; ENTER Secondary (OR 1.2 [95% CI: 0.4-4.2] p=0.7479)	7

Authors, year, country	Study type	Sample in the analysis	Caries index	Effect measure	Income: categories of analysis Socioeconomic parameter (reference*)	Group with higher caries index	Bivariate Analysis p value	Multivariate analysis p value	NOS
								Primary (OR 1.7 [95%CI: 0.4-7.3] p=0.4554)	
Gao et al. (2018) China	Cross-sectional, national survey	35-44 age group: 4410 participants (2197 males, 2213 females) 65-74 age group: 4431 participants (2222 males, 2209 females)	DR (Decayed Roots); FR (Filled Roots without primary or secondary caries); RCI (Root Caries Index - number of teeth with root caries lesions / number of teeth with gingival recession x 100%)	Prevalence, means (SD); OR	low, medium, high (reference not direct specified)	not direct specified	p values not reported	High educational level 35-44-y-old (OR 0.63 [95%CI 0.56-0.71] p<0.001) 65-74-y-old (OR 0.81 [95%CI 0.74-0.89] p<0.001)	6
Zanella-Calzada et al. (2018) United States	Case-control	9812 participants from NHANES 2013-2014 (6122 cases with dental caries, 3690 controls without caries) split across three age groups, excluding 0-9 year olds due to lack of data: Group 2 (10-19 years): 112 subjects (50 cases, 62 controls) Group 3 (20-59 years): 7603 subjects (4551 cases, 3052 controls) Group 4 (60+ years): 2097 subjects (1521 cases, 576 controls)	Presence/absence of dental caries (binary: 1 = caries present, 0 = caries absent)	OR	Highest grade or level of school completed, and household reference person's spouse's education level.		NA	**Age Group 3** Highest grade or level of school completed or the highest degree received (OR 1.340 [CI 2.5%-97.5%: 1.270 – 1.414]) HH reference person’s spouse’s education level (OR 0.837 [CI 2.5%-97.5%: 0.789 – 0.888]) **Age Group 4** Highest grade or level of school completed or the highest degree received (OR 1.471 [CI 2.5%-97.5%: 1.339 – 1,618])	7

Authors, year, country	Study type	Sample in the analysis	Caries index	Effect measure	Income: categories of analysis Socioeconomic parameter (reference*)	Group with higher caries index	Bivariate Analysis p value	Multivariate analysis p value	NOS
Amornsuradech; Vejvithee (2019) Thailand	Cross-sectional	1518 adults aged 35-44 years from the 7th Thailand National Oral Health Survey (2012)	Number of teeth with untreated dental caries (0 or ≥1).	OR	Primary complete or less; (At least secondary complete*)	Primary complete or less	<0.001	(OR 1.970 [95%CI: not reported]; p= 0.007) Model: SES variables adjusted for income, education, occupation, personal background, behavioral and access.	6
Nogueira et al. (2019), Brazil	Cross-sectional	6051 adults aged 35-44 years	DMFT index (dichotomized by the median into <16 and >16), number of decayed teeth (dichotomized by the median into 0 and >0), and number of missing teeth (dichotomized by the median into <4 and >4).	OR	Up to 8 years of study and (> 8 years of study*)	up to 8 years of study	Not reported	Decayed>0 (OR 1.32 [95%CI 1.12–1.56]) Missing>4 (OR 2.13 [95%CI 1.90–2.38]) DMFT>16 (OR 1.51 [95%CI 1.35–1.69])	7
Singla et al. (2020) India	Cross-sectional	780 adults aged 20-50 years-old	DMF-T; D-T; M-T; F-T	Mean (SD) Relative Risk (RR)	Primary school; Middle school; High school; P.U.C/diploma; (Graduate/postgraduate*)	Graduate/postgraduate	Not reported	Primary school (RR 0.80 [95%CI: 0.70-0.91] p=0.001) Middle school (RR 1.02 [95%CI: 0.91-1.14] p=0.05) High school (RR 0.90 [95%CI: 0.82-1.0] p=0.04) P.U.C/diploma (RR 0.88 [95%CI: 0.80-1.0] p=0.008)	7
Beenackers et al. (2021), Netherlands	Secondary data analysis of a cross-sectional survey conducted in 2013	452 dentate adults aged 25-44 years (combined sample of 217 aged 25-34 and 235 aged 35-44)	DMF-S. Caries were assessed at the dentine threshold (D3).	OR; RR	Low education; (High education*)	Not reported	Not reported	Negative binomial hurdle models for caries experience (having a DMFS >0 versus DMFS = 0) **MODEL 1** (The first model explored the association between	6

Authors, year, country	Study type	Sample in the analysis	Caries index	Effect measure	Income: categories of analysis Socioeconomic parameter (reference*)	Group with higher caries index	Bivariate Analysis p value	Multivariate analysis p value	NOS
								financial strain and DMFS - adjusted for age, sex, educational level, and income) Low educated (OR 5.57 [95%CI:1.55-20.0])<br><br>Negative binomial hurdle models for caries experience (amount of DMFS in those having a DMFS >0)<br>**MODEL 1** (The first model explored the association between financial strain and DMFS- adjusted for age, sex, educational level, and income) Low educated (RR 1.55 [95%CI: 1.23-1.94])	
Jouhar et al. (2021) Saudi Arabia	Cross-sectional	185 male dental students and interns	DMF-T; D-T; M-T; F-T	OR	Father and mother education: No Education; School level; College level; University level. For the regression models: No; (Yes*)	Not reported	Not reported	**Father education**<br>Decayed (Yes/No) (OR: 3.38 [95%CI 1.53-7.79])<br><br>Missing (Yes/No) (OR: 1.14 [95%CI 0.05-2.49])<br><br>Filled (Yes/No) (OR: 1.81 [95%CI 0.75-4.33])<br><br>**Mother education**<br>Decayed (Yes/No) (OR: 0.89 [95%CI 0.68-1.16])<br><br>Missing (Yes/No) (OR: 6.60 [95%CI	5

Authors, year, country	Study type	Sample in the analysis	Caries index	Effect measure	Income: categories of analysis Socioeconomic parameter (reference*)	Group with higher caries index	Bivariate Analysis p value	Multivariate analysis p value	NOS
								0.36-1.28])<br><br>Filled (Yes/No)<br>(OR: 0.67 [95%CI 0.29-1.54])	
Abozor; Abduljawad (2022) Saudi Arabia	Cross-sectional	317 adult dental patients	number of teeth having identifiable periapical lesions (PL); root-filled teeth (RFT), and number of RFT having identifiable PL	Beta	No education; primary; middle; high school; college	Root filled teeth - no education 27.6%<br>Periapical lesion – high school 27.8%	Root filled teeth p=0.040<br>Periapical lesion p=0.08	Root filled teeth Standardized coefficient, Beta -0.56; T -1.219; p=0.224<br><br>Periapical lesion Education was not included in the model	7
Almario-Barrera; Concha-Sánchez (2022) Colombia	Cross-sectional	84 working women at a private university in Santander, Colombia	Presence/Absence of dental caries	Prevalence	Primary; secondary; technical; technology; university; specialization; master's	Within those with dental caries: Technical (n=17; 23.6%)	p=0.414	Not performed	6
Stangvaltaite-Mouhat et al. (2023) Lithuania	Repeated cross-sectional national surveys	1997/1998: 569 individuals (273 adults 35-44, 296 early elderly 65-74).<br><br>2017/2019: 723 individuals (353 adults 35-44, 370 early elderly 65-74).	Decayed Surfaces (DS), Missing Teeth (MT), and DMF-T	Incidence Rate Ratios (IRR)	(Secondary school or less*); more than secondary school	Not reported	Not reported	DS<br>35-44 years-old – IRR 0.50 (95%CI: 0.39-0.64)<br>65-74 years-old - IRR 1.01 (95%CI: 0.68-1.51)<br><br>MT<br>35-44 years-old – IRR 0.53 (95%CI: 0.43-0.65)<br>65-74 years-old - IRR 0.79 (95%CI: 0.71-0.88)<br><br>DMFT<br>35-44 years-old – IRR 0.95 (95%CI: 0.88-1.03)<br>65-74 years-old - IRR 0.95 (95%CI: 0.80-1.00)	6

Authors, year, country	Study type	Sample in the analysis	Caries index	Effect measure	Income: categories of analysis Socioeconomic parameter (reference*)	Group with higher caries index	Bivariate Analysis p value	Multivariate analysis p value	NOS
Tahani et al. (2023) Iran	Ecological	Aggregated data from all 31 provinces of Iran from the 2011 Iranian Oral Health Survey (n not reported)	DMF-T; D-T; M-T; F-T; Edentulousness	Beta	Mean years of schooling of adults aged 25+; Expected years of schooling of children aged 6	Not reported	Edentulousness Mean Years of Schooling (Beta -1.01; std error 0.63 [95%CI: -2.31 - -0.28] r= -0.28; R2 8%. p>0.005) Expected years of schooling (Beta -0.18; std error 0.78 [95%CI: -1.7 - -1.4] r= -0.04; R2 0.2%%. p>0.005)<br><br>DMF-T Mean Years of Schooling (Beta -0.31; std error 0.32 [95%CI: -0.96 - 0.36] r= -0.16; R2 2%. p>0.005) Expected years of schooling (Beta -0.00; std error 0.39 [95%CI: -0.8 - 0.79] r= -0.00; R2 0.1. p>0.005)	Edentulousness Mean years of Schooling (Beta -1.08 [95%CI: -2.04 - -1.2] p=0.0029)<br><br>DT Expected years of schooling (Beta -0.62 [95%CI: -1.07 - -0.17] p=0.009)<br><br>FT Mean years of Schooling (Beta 0.56 [95%CI 0.24 - 0.89] p=0.002)	5
Orhan et al (2024) Turkey	Cross sectional	2390 adults aged 35-44	DMFT>0 D>0 M>0 F>0	percentage, PR (95%CI)	Education: high, middle, low*	low	Non information	log-binomial model or robust Poisson regression model DMFT>0 middle PR= 1.000 ((0.97–1.02) high DMFT>0 PR= 1.01 (0.99–1.04)	6

Risk of bias was assessed using the Newcastle-Ottawa (NOS) for observational studies (Wells et al. 2009) with scores for summarizing the multitude data. DMFT decayed missing filled teeth; DT decayed teeth; DS/DFS decayed (filled) surfaces; DMFS decayed missing filled surfaces; RDFS, RDS decayed (filled) surfaces root; DF decayed root; Mean (SD) standard deviation; (SE) standard error; RR risk ratio; 95%CI confidence interval; OR odds ratio; PR prevalence rate; R correlation coefficient.

Supplementary Table S3. Study characteristics and results reported of the occupational status.

Authors, year, country	Study type	Sample in the analysis	Caries index	Effect measure	Income: categories of analysis Socioeconomic parameter (reference*)	Group with higher caries index	Bivariate Analysis p value	Multivariate analysis p value	NOS
Arrica et al. (2017) Italy	Cross-sectional	480 adults (52.9% men, 47.1% women), aged 30-45 years	DMFT and FS-T (Filled and Sound Teeth)	Mean (SD) Relative Risk Ratio (RRR), SE, (95% CI)	(unemployed/housewife*) vs. technician/clerk/professional	technician/ clerk/ professional	DMF-T (One-way ANOVA: F=2.52 p<0.01)	0–5 DMF-T (base outcome); 6-10 DMF-T (RRR 1.67 [95% CI: 1.28–2.17] p<0.001); 10-15 DMF-T (RRR 2.26 [95% CI: 1.67–3.04] p<0.001); 15-28 DMF-T (RRR 3.19 [95%CI: 2.10–4.84] p<0.001)	8
Amornsuradech; Vejvithee (2019) Thailand	Cross-sectional	1518 adults aged 35-44 years from the 7th Thailand National Oral Health Survey (2012)	Number of teeth with untreated dental caries (0 or ≥1).	OR	Personal Business; Wage-earner/freelance; Agriculture; Housekeeper; (Others*)	Agriculture	Personal Business (p=0.007); Wage-earner/freelance (p<0.001); Agriculture (p=0.165); Housekeeper (p=0.007); others (ref)	Personal Business (OR: 0.828 [95%CI not reported] p=0.649); Wage-earner/freelance (OR: 1.119 [95%CI not reported] p=0.749); Agriculture (OR: 0.594 [95%CI not reported] p=0.152); Housekeeper (OR: 0.933 [95%CI not reported] p=0.877) Model: SES variables adjusted for income, education, occupation, personal background, behavioral and access.	6
Singla et al. (2020) India	Cross-sectional	780 adults aged 20-50 years-old	DMF-T; D-T; M-T; F-T	Mean (SD) Relative Risk (RR)	Unemployed; Unskilled; Skilled/semiskilled; Clerical, farmer, and business; (Semiprofessional/professional*)	Semiprofessional/ professional	Not reported	Unemployed (RR 1.27 [95%CI: 0.97-1.67] p=0.008) Unskilled (RR 1.37 [95%CI: 1.04-1.82] p=0.03) Skilled/semiskilled	7

Authors, year, country	Study type	Sample in the analysis	Caries index	Effect measure	Income: categories of analysis Socioeconomic parameter (reference*)	Group with higher caries index	Bivariate Analysis p value	Multivariate analysis p value	NOS
								(RR 0.99 [95%CI: 0.75-1.30] p=0.94) Clerical, farmer, and business (RR 0.90 [95%CI: 0.69-1.17] p=0.42)	
Jouhar et al. (2021) Saudi Arabia	Cross-sectional	185 male dental students and interns	DMF-T; D-T; M-T; F-T	OR	Father and mother occupation: Government Service; Private Service; Business; Others. For the regression models: No; (Yes*)	Not reported	Not reported	**Father occupation** Decayed (Yes/No) (OR: 0.92 [95%CI 0.65-1.31])<br><br>Missing (Yes/No) (OR: 1.18 [95%CI 0.86-1.61])<br><br>Filled (Yes/No) (OR: 0.73 [95%CI 0.51-1.05])<br><br>**Mother occupation** Decayed (Yes/No) (OR: 0.59 [95%CI 0.28-1.23])<br><br>Missing (Yes/No) (OR: 0.28 [95%CI 0.17-0.62])<br><br>Filled (Yes/No) (OR: 0.45 [95%CI 0.19-0.92])	5
Almario-Barrera; Concha-Sánchez (2022) Colombia	Cross-sectional	84 working women at a private university in Santander, Colombia	Presence/Absence of dental caries	Prevalence	Occupation at the university<br>Professor<br>Business<br>General services<br>Learner SENA<br><br>Working hours<br>Full-time<br>Half-time<br>Catedra<br><br>Solely work at the university	Within those with dental caries: Occupation at the university Business (n= 31; 43.1%)<br><br>Working hours Full-time (n=65; 90.3%)<br><br>Solely work at the	Occupation at the university p=0.635<br><br>Working hours p=1.000<br><br>Solely work at the university p= 0.167	Not performed	6

Authors, year, country	Study type	Sample in the analysis	Caries index	Effect measure	Income: categories of analysis Socioeconomic parameter (reference*)	Group with higher caries index	Bivariate Analysis p value	Multivariate analysis p value	NOS
					Yes	university<br>Yes (n=55; 76.4%)			
Orhan et al (2024) Turkey	Cross-sectional	2390 adults aged 35-44	DMFT>0<br>D>0<br>M>0<br>F>0	Percentage, PR (95%CI)	Employment status: No	yes	No information	log-binomial model or robust Poisson regression model based<br><br>DMFT>0<br>PR= 1.00 (0.98–1.03)	6

Risk of bias was assessed using the Newcastle-Ottawa (NOS) for observational studies (Wells et al. 2009) with scores for summarizing the multitude data. DMFT decayed missing filled teeth; DT decayed teeth; DS/DFS decayed (filled) surfaces; DMFS decayed missing filled surfaces; RDFS, RDS decayed (filled) surfaces root; DF decayed root; Mean (SD) standard deviation; (SE) standard error; RR risk ratio; 95%CI confidence interval; OR odds ratio; PR prevalence rate; R correlation coefficient.

Supplementary Table S4. Study characteristics and results reported of the socioeconomic status.

Authors, year, country	Study type	Sample in the analysis	Caries index	Effect measure	Income: categories of analysis Socioeconomic parameter (reference*)	Group with higher caries index	Bivariate Analysis p value	Multivariate analysis p value	NOS
Gijwani et al. (2020) India	Cross-sectional	995 adults aged 35-44	DMF-T; D-T; M-T; F-T	Mean; SD	Kuppuswamy's Scale for SES (upper, upper middle, lower middle, upper lower, lower)	Higher mean DMF-T scores in the upper lower group (6.99; SD 3.213)	0.01	Not conducted	7
Singla et al. (2020) India	Cross-sectional	780 adults aged 20-50 years-old	DMF-T; D-T; M-T; F-T	Mean (SD) Relative Risk (RR)	Kuppuswamy's Scale for SES: Lower, Upper lower; Lower middle; Upper middle; (Upper*)	Upper	Not reported	Lower (RR 0.71[95%CI: 0.43-1.17] p<0.001)<br>Upper lower (RR 0.65 [95%CI: 0.40-1.05] p=0.01)<br>Lower middle (RR 0.92 [95%CI: 0.60-1.43] p=0.72)<br>Upper middle (RR 0.84 [95%CI: 0.60-1.17] p=0.3)	7
Almario-Barrera; Concha-Sánchez (2022) Colombia	Cross-sectional	84 working women at a private university in Santander, Colombia	Presence/Absence of dental caries	Prevalence	Socioeconomic strata<br>Two<br>Three<br>Four<br>Five<br>Six<br><br>Socioeconomic position<br>Low<br>Media<br>High	Within those with dental caries:<br>Socioeconomic strata<br>Three (n= 29; 40.3%) p=0.309<br><br>Socioeconomic position<br>Low (n=30; 41.7%) p=0.246	Socioeconomic strata p=0.309<br><br>Socioeconomic position p=0.246	Not performed	6
Bukmir et al. (2022) Croatia	Cross-sectional	597 participants – 190 males (31.8%) and 407 females (68.2%)	D-T	Beta	Self-assessed economic status of household: below the average; average; above the average	Not reported	Not reported	Self-assessed economic status of household (B −1.296; SE 0.299; p<0.001; Sr−0.177). R2 adj=0.341	7
Celeste et al. (2024) Brazil	Cross-sectional analysis of a prospective cohort study (Epi-Floripa Study, second wave)	1099 individuals aged 20-59	DMF-T. The number of missing teeth (MT) and untreated dental caries (DT) were dichotomized as MT >0 and DT >0	Prevalence, prevalence ratios (PRs), and Relative Excess Risk due to Interaction (RERI)	Social mobility: Persistently lower; Upwardly mobile; Downwardly mobile; Persistently higher	MT>0 (Persistent lower, prevalence 86.7%)<br>DT>0 (Downwardly mobile, prevalence 35.0%)	p<0.01	Not performed for caries outcomes. RERI and Oaxaca-Blinder decomposition analyses were conducted to assess effect modification and mediation by dental care variables.	6

Risk of bias was assessed using the Newcastle-Ottawa (NOS) for observacional studies (Wells et al. 2009) with scores for summarizing the multitude data. DMFT decayed missing filled teeth; DT decayed teeth; DS/DFS decayed (filled) surfaces; DMFS decayed missing filled surfaces; RDFS, RDS decayed (filled) surfaces root; DF decayed root; Mean (SD) standard deviation; (SE) standard error; RR risk ratio; 95%CI confidence interval; OR odds ratio; PR prevalence rate; R correlation coefficient.

Supplementary Table S5. Study characteristics and results reported of the collective indicators and other.

Authors, year, country	Study type	Sample in the analysis	Caries index	Effect measure	Income: categories of analysis Socioeconomic parameter (reference*)	Group with higher caries index	Bivariate Analysis p value	Multivariate analysis p value	NOS
Oscarson; Espelid; Jönsson (2017) Norway	Cross-sectional	932 adults (988 women) with at least one tooth, mean age 47.0 years (SD 15.3)	DMF-S, DF-S, DMF-T, DF-T, DT, Number-T Intact-T. DS1-5 (Sum of enamel and dentine caries) DS1-2 (Enamel caries) DS3-5 (Dentine caries)	Mean caries scores, odds ratios (OR)	Municipality: Urban, Suburban, Rural (note: divided into three categories based on the number of inhabitants and dentists per inhabitants)	Rural (Mean number of surfaces with primary caries: 5.0 [SD 6.7)	p<0.001	Not included in tha analysis	7
Amornsuradech; Vejvithee (2019) Thailand	Cross-sectional	1518 adults aged 35-44 years from the 7th Thailand National Oral Health Survey (2012)	Number of teeth with untreated dental caries (0 or ≥1).	OR	Area of residence: Bangkok, other urban, (rural*); Region of residence: Central, North, Northeast, South, (Bangkok*)	Rural; Northeast	>0.005	Area of residence: Bangkok (OR: 0.545 [95%CI not reported] p=0.152) Other urban (OR: 0.926 [95%CI not reported] p=0.745). Region of residence: Central (OR: 0.615 [95%CI not reported] p=0.104), North (OR 0.438 [95%CI not reported] p=0.022), Northeast (OR: 0.458 [95%CI not reported] p=0.006), South (N/C, not calculated due to automatic exclusion during analysis) Model: SES variables adjusted for income, education, occupation, personal background, behavioral and access.	6
Zurabiani et al. (2019) Georgia	Cross-sectional	1027 adults (across five age groups: 35-44, 45-54, 55-64, 65-74, 75-84)	DMF-T	Mean DMF-T scores, prevalence	Regions: Svaneti, Racha, Samegrelo, Tbilisi, Kakheti, Tianeti	Higher DMF-T scores in Kakheti (12.56; SD 8.27)	<0.05	Not conducted	5
Singla et al. (2020) India	Cross-sectional	780 adults aged 20-50 years-old	DMF-T; D-T; M-T; F-T	Mean (SD) Relative Risk (RR)	Rural; (Urban*)	Urban	Not reported	Rual (RR 1.00 [95%CI: 0.92-1.83] p=0.975)	7
Almario-Barrera; Concha-Sánchez (2022) Colombia	Cross-sectional	84 working women at a private university in Santander, Colombia	Presence/Absence of dental caries	Prevalence	Rural; Urban	Within those with dental caries: Urban (n=71; 98.6%)	Urban p=0.267	Not performed	6
Bukmir et al. (2022) Croatia	Cross-sectional	597 participants – 190 males (31.8%) and 407 females (68.2%)	D-T	Beta	Rural; Urban	Not reported	Not reported	Residency (B−2.008; SE 0.380; p<0.001; Sr −0.213). R2 adj=0.341	7

Authors, year, country	Study type	Sample in the analysis	Caries index	Effect measure	Income: categories of analysis Socioeconomic parameter (reference*)	Group with higher caries index	Bivariate Analysis p value	Multivariate analysis p value	NOS
Stangvaltaite-Mouhat et al. (2023) Lithuania	Repeated cross-sectional national surveys	1997/1998: 569 individuals (273 adults 35-44, 296 early elderly 65-74).<br><br>2017/2019: 723 individuals (353 adults 35-44).	Decayed Surfaces (DS), Missing Teeth (MT), and DMF-T	Incidence Rate Ratios (IRR)	(Urban*); Rural	Not reported	Not reported	DS<br>35-44 years-old – IRR 1.26 (95%CI: 0.98-1.62)<br>65-74 years-old - IRR 1.23 (95%CI: 0.88-1.72)<br><br>MT<br>35-44 years-old – IRR 1.32 (95%CI: 1.09-1.60)<br>65-74 years-old - IRR 1.06 (95%CI: 0.94-1.20)<br><br>DMFT<br>35-44 years-old – IRR 0.98 (95%CI: 0.91-1.06)<br>65-74 years-old - IRR 0.99 (95%CI: 0.94-1.05)	6
Tahani et al. (2023) Iran	Ecological	Aggregated data from all 31 provinces of Iran from the 2011 Iranian Oral Health Survey (n not reported)	DMF-T; D-T; M-T; F-T; Edentulousness	Beta	Gross National Income (GNI) per capita	Not reported		Simple linear regression<br>Edentulousness<br>GNI (Beta 0.12; std error 0.25 [95%CI: -0.39 - 0.64] r=0.09; R2 0.8%. p>0.005)<br><br>DMF-T<br>GNI (Beta -0.001; std error 0.13 [95%CI -0.27 - -0.24] r=-0.02; R2 0.1%. p>0.005)<br><br>GNI did not enter in the Stepwise multiple linear regression	5
Chaudhary et al. (2024) Worldwide	Ecological	Data from 170 countries worldwide (53 member countries (MC) and 117 non-member countries (n-MC) of the Organization of Islamic Cooperation (OIC)). 25 countries were excluded due to lack of data	DMF-T	Mean difference in DMF-T scores, Beta	National Income per capita (GNI): low-income (LIC); lower-middle-income (LMIC); upper-middle-income (UMIC); and high-income countries (HIC)	35-44 years-old<br>Non-Muslim countries - High income (Mean DMF-T 14.2 [SD 3.6])<br>Muslin countries - Upper Middle (Mean DMF-10.5 [SD 4.0])	p=0.004	GNI was used just for adjusting the simple linear regression model	7

Risk of bias was assessed using the Newcastle-Ottawa (NOS) for observational studies (Wells et al. 2009) with scores for summarizing the multitude data. DMFT decayed missing

filled teeth; DT decayed teeth; 95%CI confidence interval; OR odds ratio; R correlation coefficient, GDP Gross domestic product; GNI Gross national income; Gini – index used to measure inequality of income distribution.

Section and Topic	Item #	Checklist item	Location where item is reported
**TITLE**			
Title	1	Identify the report as a systematic review.	1
**ABSTRACT**			
Abstract	2	See the PRISMA 2020 for Abstracts checklist.	1, 2
**INTRODUCTION**			
Rationale	3	Describe the rationale for the review in the context of existing knowledge.	2
Objectives	4	Provide an explicit statement of the objective(s) or question(s) the review addresses.	2
**METHODS**			
Eligibility criteria	5	Specify the inclusion and exclusion criteria for the review and how studies were grouped for the syntheses.	3
Information sources	6	Specify all databases, registers, websites, organisations, reference lists and other sources searched or consulted to identify studies. Specify the date when each source was last searched or consulted.	3
Search strategy	7	Present the full search strategies for all databases, registers and websites, including any filters and limits used.	Supp File S2
Selection process	8	Specify the methods used to decide whether a study met the inclusion criteria of the review, including how many reviewers screened each record and each report retrieved, whether they worked independently, and if applicable, details of automation tools used in the process.	3
Data collection process	9	Specify the methods used to collect data from reports, including how many reviewers collected data from each report, whether they worked independently, any processes for obtaining or confirming data from study investigators, and if applicable, details of automation tools used in the process.	3, 4
Data items	10a	List and define all outcomes for which data were sought. Specify whether all results that were compatible with each outcome domain in each study were sought (e.g. for all measures, time points, analyses), and if not, the methods used to decide which results to collect.	3, 4
	10b	List and define all other variables for which data were sought (e.g. participant and intervention characteristics, funding sources). Describe any assumptions made about any missing or unclear information.	3, 4
Study risk of bias assessment	11	Specify the methods used to assess risk of bias in the included studies, including details of the tool(s) used, how many reviewers assessed each study and whether they worked independently, and if applicable, details of automation tools used in the process.	4
Effect measures	12	Specify for each outcome the effect measure(s) (e.g. risk ratio, mean difference) used in the synthesis or presentation of results.	4
Synthesis methods	13a	Describe the processes used to decide which studies were eligible for each synthesis (e.g. tabulating the study intervention characteristics and comparing against the planned groups for each synthesis (item #5)).	4
	13b	Describe any methods required to prepare the data for presentation or synthesis, such as handling of missing summary statistics, or data conversions.	NA
	13c	Describe any methods used to tabulate or visually display results of individual studies and syntheses.	4
	13d	Describe any methods used to synthesize results and provide a rationale for the choice(s). If meta-analysis was performed, describe the model(s), method(s) to identify the presence and extent of statistical heterogeneity, and software package(s) used.	4
	13e	Describe any methods used to explore possible causes of heterogeneity among study results (e.g. subgroup analysis, meta-regression).	NA
	13f	Describe any sensitivity analyses conducted to assess robustness of the synthesized results.	NA

Section and Topic	Item #	Checklist item	Location where item is reported
Reporting bias assessment	14	Describe any methods used to assess risk of bias due to missing results in a synthesis (arising from reporting biases).	4
Certainty assessment	15	Describe any methods used to assess certainty (or confidence) in the body of evidence for an outcome.	NA
**RESULTS**			
Study selection	16a	Describe the results of the search and selection process, from the number of records identified in the search to the number of studies included in the review, ideally using a flow diagram.	4, 5<br>Fig 1
	16b	Cite studies that might appear to meet the inclusion criteria, but which were excluded, and explain why they were excluded.	Fig 1
Study characteristics	17	Cite each included study and present its characteristics.	4<br>Sup. Tables S1-S5
Risk of bias in studies	18	Present assessments of risk of bias for each included study.	5<br>Sup. Tables S1-S5
Results of individual studies	19	For all outcomes, present, for each study: (a) summary statistics for each group (where appropriate) and (b) an effect estimate and its precision (e.g. confidence/credible interval), ideally using structured tables or plots.	Sup. Tables S1-S5
Results of syntheses	20a	For each synthesis, briefly summarise the characteristics and risk of bias among contributing studies.	5, 9<br>Sup. Tables S1-S5
	20b	Present results of all statistical syntheses conducted. If meta-analysis was done, present for each the summary estimate and its precision (e.g. confidence/credible interval) and measures of statistical heterogeneity. If comparing groups, describe the direction of the effect.	NA
	20c	Present results of all investigations of possible causes of heterogeneity among study results.	4, 6-8
	20d	Present results of all sensitivity analyses conducted to assess the robustness of the synthesized results.	NA
Reporting biases	21	Present assessments of risk of bias due to missing results (arising from reporting biases) for each synthesis assessed.	NA
Certainty of evidence	22	Present assessments of certainty (or confidence) in the body of evidence for each outcome assessed.	NA
**DISCUSSION**			
Discussion	23a	Provide a general interpretation of the results in the context of other evidence.	8
	23b	Discuss any limitations of the evidence included in the review.	9
	23c	Discuss any limitations of the review processes used.	9
	23d	Discuss implications of the results for practice, policy, and future research.	10
**OTHER INFORMATION**			
Registration and protocol	24a	Provide registration information for the review, including register name and registration number, or state that the review was not registered.	3
	24b	Indicate where the review protocol can be accessed, or state that a protocol was not prepared.	3
	24c	Describe and explain any amendments to information provided at registration or in the protocol.	NA

Section and Topic	Item #	Checklist item	Location where item is reported
Support	25	Describe sources of financial or non-financial support for the review, and the role of the funders or sponsors in the review.	10
Competing interests	26	Declare any competing interests of review authors.	10
Availability of data, code and other materials	27	Report which of the following are publicly available and where they can be found: template data collection forms; data extracted from included studies; data used for all analyses; analytic code; any other materials used in the review.	11

From: Page MJ, McKenzie JE, Bossuyt PM, Boutron I, Hoffmann TC, Mulrow CD, et al. The PRISMA 2020 statement: an updated guideline for reporting systematic reviews. BMJ 2021;372:n71. doi: 10.1136/bmj.n71. This work is licensed under CC BY 4.0. To view a copy of this license, visit https://creativecommons.org/licenses/by/4.0/

Supplementary File S2 - Search strategy for each electronic database, data May 05, 2024

Database	Search strategy
MEDLINE (PubMed)	(caries OR Dental Caries OR dental decay OR DMF index OR decayed teeth OR DMFS OR DMFT) AND (socioeconomic factors OR social class OR educational status OR educational level OR socioeconomic condition OR socioeconomic level OR socioeconomic determinant* OR social determinant* OR income OR poverty OR risk factors OR occupational class) – Limited to include studies with subjects ≥19 years old, published between 2017 to 2024
Cochrane Library	#1 caries<br>#2 MeSH descriptor: [Dental Caries] explode all trees<br>#3 dental decay<br>#4 MeSH descriptor: [DMF Index] explode all trees<br>#5 decayed teeth<br>#6 #1 or #2 or #3 or #4 or #5<br>#7 MeSH descriptor: [Socioeconomic Factors] explode all trees<br>#8 MeSH descriptor: [Social Class] explode all trees<br>#9 MeSH descriptor: [Educational Status] explode all trees<br>#10 educational level<br>#11 socioeconomic condition<br>#12 socioeconomic level<br>#13 socioeconomic determinant*<br>#14 social determinant*<br>#15 MeSH descriptor: [Income] explode all trees<br>#16 MeSH descriptor: [Poverty] explode all trees<br>#17 MeSH descriptor: [Risk Factors] explode all trees<br>#18 #7 or #8 or #9 or #10 or #11 or #12 or #13 or #14 or #15 or #16 or #17<br>#19 #6 and #18 with Cochrane Library publication date between Mar 2017 and Apr 2024
Web of Science	(caries OR Dental Caries OR dental decay OR DMF index OR decayed teeth OR DMFS OR DMFT) AND (socioeconomic factors OR social class OR educational status OR educational level OR socioeconomic condition OR socioeconomic level OR socioeconomic determinant* OR social determinant* OR income OR poverty OR risk factors OR occupational class) - Limited to include studies published between 2017 to 2024
Controlled-Trials Database	Each keyword was used separately:<br>Dental decay<br>Cavitated teeth

ClinicalTrials.gov (U.S. National Institutes of Health)	Dental caries AND socioeconomic factors
The National Institute for Health and Care Excellence	Each keyword was used separately:<br>Dental decay<br>Cavitated teeth
